# Graphene Growth Directly on SiO_2_/Si by Hot Filament Chemical Vapor Deposition

**DOI:** 10.3390/nano12010109

**Published:** 2021-12-30

**Authors:** Sandra Rodríguez-Villanueva, Frank Mendoza, Alvaro A. Instan, Ram S. Katiyar, Brad R. Weiner, Gerardo Morell

**Affiliations:** 1 Department of Physics, College of Natural Science, Rio Piedras Campus, University of Puerto Rico, San Juan, PR 00925, USA; alvaro.instan@upr.edu (A.A.I.); ram.katiyar@upr.edu (R.S.K.); brad.weiner@upr.edu (B.R.W.); gerardo.morell@upr.edu (G.M.); 2 Molecular Sciences Research Center, University of Puerto Rico, San Juan, PR 00927, USA; 3 Department of Physics, College of Arts and Sciences, Mayagüez Campus, University of Puerto Rico, Mayaguez, PR 00682, USA; fran.mendoza@upr.edu; 4 Department of Chemistry, College of Natural Science, Rio Piedras Campus, University of Puerto Rico, San Juan, PR 00925, USA

**Keywords:** graphene, hot filament chemical vapor deposition, copper catalytic effect

## Abstract

We report the first direct synthesis of graphene on SiO_2_/Si by hot-filament chemical vapor deposition. Graphene deposition was conducted at low pressures (35 Torr) with a mixture of methane/hydrogen and a substrate temperature of 970 °C followed by spontaneous cooling to room temperature. A thin copper-strip was deposited in the middle of the SiO_2_/Si substrate as catalytic material. Raman spectroscopy mapping and atomic force microscopy measurements indicate the growth of few-layers of graphene over the entire SiO_2_/Si substrate, far beyond the thin copper-strip, while X-ray photoelectron spectroscopy and energy-dispersive X-ray spectroscopy showed negligible amounts of copper next to the initially deposited strip. The scale of the graphene nanocrystal was estimated by Raman spectroscopy and scanning electron microscopy.

## 1. Introduction

Since graphene was first obtained by microexfoliation of graphite in 2004 [1] it has been regarded as a promising material due to its excellent properties and potential applications [2,3,4,5,6,7]. Graphene’s high electron mobility, conductivity, and optical properties open up the possibility for high-speed electronics such as ultra-thin transistors, photodetectors, and optical modulators [2,3]. These attributes also contribute to the advancement of circuit boards, display panels, and solar cell technology [2,3], while its high internal surface area, electrochemical reactivities and mechanical (high stiffness and low density) properties allow greater efficiency in supercapacitors, electrochemical systems, and strain sensors, respectively [3,4,5]. Many studies have focused on obtaining graphene using a wide variety of methods [8,9,10,11], e.g., the microexfoliation of graphite [1], graphene oxide reduction [12], epitaxial growth on SiC [13,14] and chemical vapor deposition (CVD) on different substrates [15,16]. This last method is the most promising because the growth parameters can be controlled to modify the structural characteristics of the material and the number of graphene layers (monolayer, bilayer, few layers, and multilayers) deposited [17,18]. Graphene growth by CVD on metallic substrates has been used extensively, because the catalytic properties of the substrate result in a large area of high-quality graphene [19,20,21]. In order to scale this technology to industrial production, hot filament chemical vapor deposition (HFCVD) promises to be one of the leading potential techniques. This method obtains large area, high quality graphene on copper substrates with controllable growth parameters [18,22,23,24]. The hot filament dissociates the hydrogen and methane, producing active radicals that reduce the amorphous carbon to improve the quality of the graphene film [24]. The turbulent flow produced by the vertical introduction of the gas in the HFCVD provides an additional advantage for scale-up compared with the laminar flow of a tube furnace CVD. However, for use in electronic applications, current chemical vapor deposition methods require the transfer of the graphene film from the metal substrate to the dielectric, which has several drawbacks, i.e., residual chemical contamination and the risk of wrinkling or breakage of the graphene film [25]. To avoid this difficult transfer process, researchers have sought to develop new methodologies to deposit graphene directly onto non-metallic substrates such as SiO_2_/Si, quartz, fused silica, and others [26]. To date, there are no reports in the literature of the direct deposition of graphene on SiO_2_ by HFCVD, although several attempts by tube furnace CVD have been published. Table 1 presents the different methods of graphene deposition on SiO_2_ by other types of CVD. This table summarizes these methodologies under two classifications: Catalyst-free and metal catalyzed direct growth CVD, where both regular and plasma enhanced CVD (PECVD) are used [26].

In the first methodology (catalyst-free), the majority of the graphene growth experiments on non-metallic substrates are conducted at high temperatures (1060–1100 °C) over a long deposition time [26]. Liu et al. obtained high-quality monolayer, bilayer and few-layer graphene without any catalyst over a temperature range of 1060–1100 °C at atmospheric pressure and using methane as the carbon source [27]. Sun et al. were able to grow continuous nanocrystalline graphene at 1000 °C with good electrical properties, such as sheet resistance and Hall mobility [28]. Medina and coworkers reported that the PECVD catalyst-free growth temperature can be reduced by directly growing a nanographene film on SiO_2_ at low temperatures (400 °C) by using the electron cyclotron resonance CVD (ECR-CVD) method [29].

In the metal-catalyzed direct growth method, many experiments have used a sacrificial metal layer to stimulate graphene growth. McNerny et al. deposited a nickel layer on SiO_2_/Si wafers as a catalyst, which was subsequently delaminated using adhesive tape, leaving behind the graphene layer on the substrate [30]. They obtained a continuous (>90% coverage) graphene film on the centimeter scale, consisting of micrometer order domains and ranging from monolayer to multilayer [30]. Dong et al. deposited a copper layer on SiO_2_/Si substrate to synthesize graphene using a CVD tube furnace [31]. They concluded that the copper evaporation occurred after the graphene deposition, but they observed some defects and residual copper in the graphene layer, which they removed by using an FeCl_3_ solution [31]. Similarly, Ismach et al. deposited a copper layer on a variety of substrates (quartz, sapphire, fused silica, and SiO_2_/Si) to promote graphene growth [32]. They found that the copper layer was dewed and evaporated during or after graphene deposition producing areas free of copper, but residues remained all over the substrate [32]. Kato et al. combined the metal catalytic method with rapid heating plasma CVD to obtain graphene on SiO_2_/Si [33]. They deposited a nickel film on the substrate and using a growth temperature ranging from 600–950 °C, obtained high-quality single-layer graphene sheets with hexagonal domains, suitable for the fabrication of a graphene-based field effect transistor [33].

This paper reports a novel method suitable for industrial scale-up production to directly grow high-quality graphene on SiO_2_/Si substrates by HFCVD. This technique allows the deposition of graphene over the entire substrate by using the metal-catalyzed method in a limited manner. A thin copper-strip was deposited on the middle of the SiO_2_/Si substrate allowing the methane dehydrogenation and the carbon absorption to occur and leaving the rest of the surface free of metal. Structural, morphological, and compositional analyses were made on the graphene grown on the SiO_2_/Si in areas on top of and next to the copper strip. This research targets SiO_2_/Si substrates due to their ubiquity in graphene applications, such as photodetectors, gas sensors, solar energy, and others [3]. In addition, we use a HFCVD system that has unique advantages in terms of scalability for deposition over large area substrates [34].

## 2. Materials and Methods

### 2.1. Substrate Preparation

**Figure 1:**
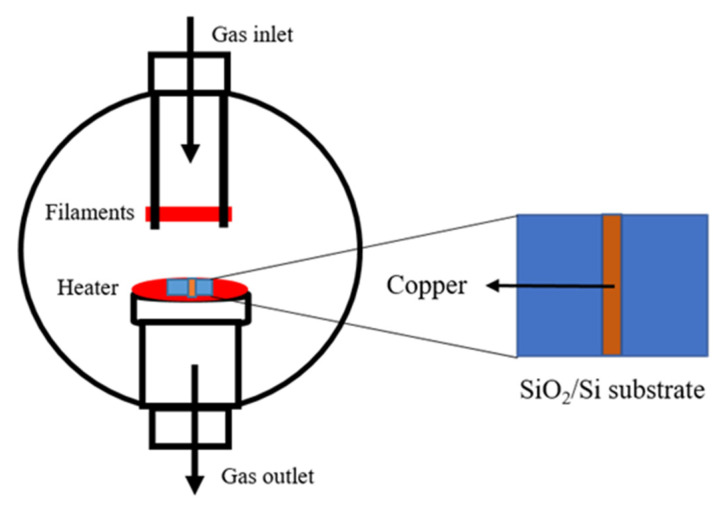
Hot filament chemical vapor deposition (HFCVD) reactor schematic and the SiO_2_/Si substrate with the deposited copper-strip.

Nanocrystalline graphene films were grown on p-type SiO_2_/Si wafers with a top oxide layer of 285 nm and a thickness of 500 ± 25 µm manufactured by Graphene Supermarket (Ronkonkoma, NY, USA; https://graphene-supermarket.com/, accessed on 26 October 2021). These wafers were cut into 2 × 2 cm pieces and cleaned with: deionized water, trichlorethylene, acetone (histology grade), and isopropanol (histology grade); the last three reagents were obtained from Fisher Scientific (Pittsburgh, PA; https://www.fishersci.com/, accessed on 26 October 2021). A mixture of sulfuric acid (H_2_SO_4_ purity range of 95–98%) and hydrogen peroxide (H_2_O_2_ solution at 30% *w*/*w* in H_2_O), both provided by Sigma Aldrich (St. Louis, MO, USA; https://www.sigmaaldrich.com/, accessed on 26 October 2021), was prepared for a further cleaning of the substrate. A thin copper-strip (3 mm width) was deposited in the middle of the SiO_2_/Si substrate by sputtering (AMNPS-1 plasma-therm, Varian, Saint Petersburg, FL, USA) with a deposition time of 1 minute (cf. Figure 1). The copper target (99.99% pure) used for the deposition was obtained from the CERAC company. The thickness of the deposited copper layer was between 100–150 nm and was measured using an Ambios Technology XP-200 profilometer (Santa Cruz, CA, USA).

### 2.2. Graphene Synthesis

A commercial HFCVD instrument (BWS-HFCVD1000, Blue Wave, Baltimore, MD, USA; https://www.bluewavesemi.com/ accessed on 26 October 2021) was used for the graphene deposition. The reactor consists of a heated substrate holder that is positioned below three heated filaments of rhenium. The gases enter the chamber from the top with a shower-like turbulent flow, (cf. Figure 1). The HFCVD instrument allows systematic adjustment of the growth parameters e.g., pressure, gas flow rates, deposition time, substrate-to-filament distance (5–15 mm), substrate temperature and filament temperature. The SiO_2_/Si substrates (4 cm^2^) with the thin copper-strip (0.3 cm × 2.0 cm) were submitted to the graphene synthesis procedure at different growth parameters. The substrate was placed in the HFCVD as shown in Figure 1, with the copper strip perpendicular with respect to the filament orientation. The pressure and heating rate were fixed at 35 Torr and 35 °C/min, respectively, for the complete process (annealing and growth steps). During the annealing stage, the substrate was kept at 975 °C with 80 sccm of hydrogen and 20 sccm of argon for 30 min.

For the growth stage, the substrate temperature was reduced to 900 °C, and the filaments were turned on at a temperature range of 1800 °C–2300 °C in an atmosphere of methane (1–10 sccm) and hydrogen (10–50 sccm) for 30 to 120 min. Finally, the samples were cooled by spontaneous convection to room temperature. As a control study, SiO_2_/Si substrates without a copper-strip were also submitted to the graphene growth procedure.

### 2.3. Characterization

The structural characterization of graphene was conducted by Raman spectroscopy (Thermo Scientific DXR, Waltham, MA, USA) equipped with an excitation laser operating at 532 nm. The spectra were collected over a frequency range of 1100 to 3100 cm^−1^ with a spot size of 0.7 μm. In addition, Raman mappings were taken over an area of 150 × 100 μm^2^ and a step size of 2 μm; the collecting time for each point in the Raman mappings was 20 s. A morphological study of the synthesized graphene was done using a scanning electron microscope, SEM (JSM 6480LV, JEOL, Peabody, MA, USA; https://www.jeol.co.jp/en/ accessed on 26 October 2021) at different magnifications (5000×, 25,000× and 140,000×) and an atomic force microscope, AFM (Nanoscope V, Vecco, Plainview, NY, USA; https://www.veeco.com/ accessed on 26 October 2021) in tapping mode over an area of 3 × 3 µm. Compositional analyses of the graphene samples were done by energy-dispersive X-ray spectroscopy, EDS (JEOL JSM 6480LV) and X-ray photoelectron spectroscopy, XPS (PHI 5600 Physical Electronics, Chanhassen, MN, USA; https://www.phi.com/index.html accessed on 26 October 2021) over an energy range of 0 to 1200 eV.

## 3. Results

A structural (Raman), morphological (SEM and AFM) and compositional (EDS and XPS) analysis was done on the synthesized graphene, both on top of and next to the copper- strip deposited in the SiO_2_/Si substrate.

### 3.1. Raman Analysis

**Figure 2:**
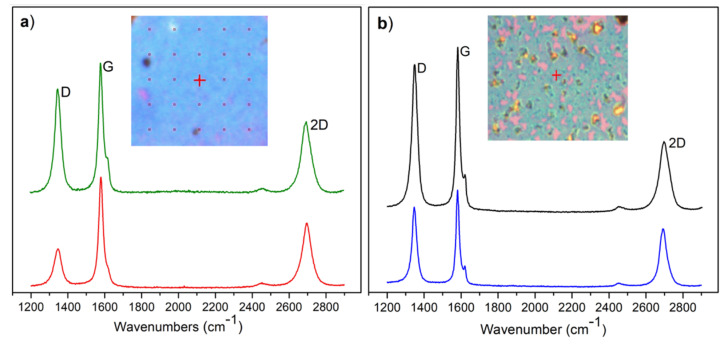
Raman spectra of graphene on SiO_2_/Si substrates and its respective peaks (D, G and 2D): (**a**) Next to the copper-strip areas and (**b**) On top of the copper-strip areas.

Characteristic of the Raman effect in graphene, the G peak is sensitive to sp^2^ carbon atoms, the 2D peak appears in response to a two-phonon vibrational process and the D peak is activated by the edges or defects in graphene [35]. All three graphene peaks were observed in the Raman spectra (cf. Figure 2), both on top of and next to the copper-strip areas on SiO_2_/Si substrate. For the control samples without a copper strip, these graphene peaks were not observed, indicating that the copper metal is necessary for the growth of graphene under our experimental conditions. Figure 2a,b show the Raman spectra on top of and next to the copper-strip area deposited on SiO_2_/Si substrate, respectively. The red and green spectra show two different signals next to the copper strip (Figure 2a) and the blue and black represent the same, but on top of the metal strip (Figure 2b). The insets show the optical images of both areas, respectively.

The G peak at 1579 cm^−1^, the 2D peak at 2692 cm^−1^ and a high D peak at 1348 cm^−1^ were observed in the Raman spectra for both areas. In addition, a peak at 1620 cm^−1^ known as D’ was found, which is related to the defects in the graphene film structure [36,37]. The D’ peak was bigger in the graphene grown on top of the copper-strip than the next to the metal film, where the peak was almost indistinguishable. This suggests that the graphene film grown on top of the copper strip has more defects. The high intensity of the D peak in both areas indicates that the carbon films are composed of nanometer-scale crystallites [36]. The presence of this peak (D) could also be associated with defects in the crystallite structure [18,37,38].

The average intensity ratio between the D and G peaks (I_D/G_) yields an estimate of the graphene grain size [39,40] and the level of the defective crystallites [36,37,41,42]. In our case, these values were between 0.30 ± 0.04 and 0.80 ± 0.03 next to the copper strip. The higher I_D/G_ values, 0.45 ± 0.07 and 0.87 ± 0.03, were found on top of the metal strip. Although, we had a significant observed D peak, the average of the full width at half maximum (FWHM) of the D, G and 2D peaks indicates good quality crystallites [36]. The FWHM of these peaks on top of the copper strip were 35 ± 1 cm^−1^, 25 ± 1 cm^−1^, and 56 ± 3 cm^−1^, respectively and in areas next to the copper strip were: 38 ± 2 cm^−1^, 29 ± 1 cm^−1^ and 52 ± 2 cm^−1^.

To calculate the crystal size from the Raman data, we employ the Cancado equation (Equation (1)) [38], where L_a_ corresponds to the crystallite size, λ_l_ represents the wavelength of the excitation laser, I_D_/I_G_ is the intensity ratio of the D and G peaks and 2.4 × 10 ^−10^ is the proportionality constant between I_D_/I_G_ and Lα. We found that the Lα on top of and next to the copper strip was in the range of 24.03 to 64.07 nm and 22.11 to 42.72 nm, respectively, in agreement with the D peak characteristics associated to the nanocrystals, but different from the grain size (35–140 nm) measured by SEM (vide infra):

(1) $$L_{\alpha }\left(\mathrm{nm}\right)=\left(2.4\times 10^{-10}\right){\;\lambda }_{l}^{4}\;{\left(I_{D}/I_{G}\right)}^{-1}$$

The difference in the particle size estimates is likely due to the multiple phonon dispersion produced by defects inside of the graphene crystallites [37,43]. These imperfections in the crystal affect the intensity ratio between the D and G peaks in the Raman spectra, resulting in false behavior of smaller grains [37,43]. To estimate the contribution of these defects, we use Equation (2) [44,45], where L_D_ represents the inter-defect distance, E_L_ is the excitation energy and the defect concentration corresponds to 1/$L_{D}^{2}$ [45]. Our results of the average L_D_ in areas next to and on top of copper strip were 18 nm and 10 nm, respectively. We also estimate the defect concentration for both areas, next to and on top of copper strip with values of 3 × 10^−3^/nm ^2^ and 7 × 10^−3^/nm ^2^, respectively. These results confirm that some point defects are present in the nanocrystals and contribute to the I_D_/I_G_ ratio intensity. In addition, we corroborate that higher concentration of defective crystals are present on top of the copper strip versus next to this metal film:

(2) $$L_{D}^{2}\;\left({\mathrm{nm}}^{2}\right)=\frac{3600}{E_{L}^{4}}{\left(I_{D}/I_{G}\right)}^{-1}$$

The G and 2D peaks characteristically correspond to the signal for graphitic materials [18], where the intensity of these peaks was higher on top of the copper-strip areas than next to this film.

**Figure 3:**
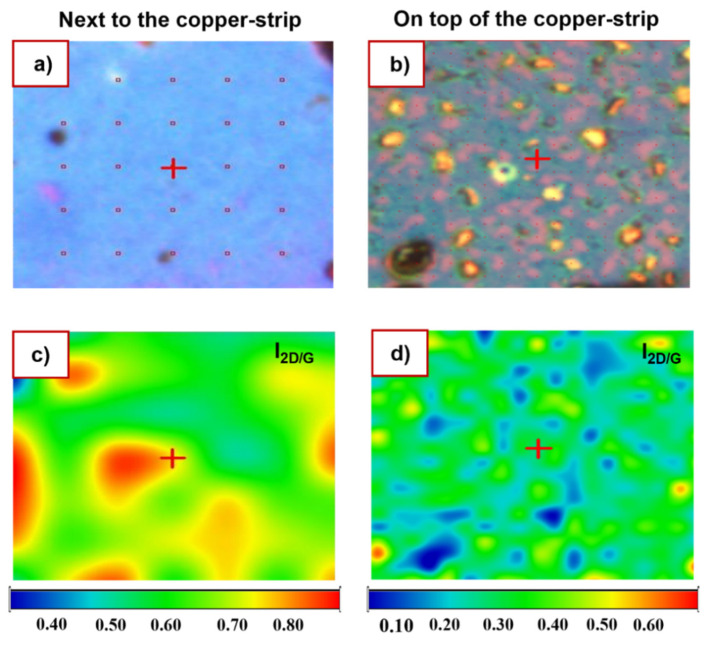
Raman mapping for graphene growth on SiO_2_/Si substrate. Where (**a**,**b**) represent the optical images of the selected mapping area (150 × 100 μm^2^) next to and on top of the copper-strip, respectively. (**c**,**d**) show the ratio between the intensity of 2D/G peaks in the same areas as in (**a**,**b**).

Raman mapping (cf. Figure 3) was done to understand the uniformity of graphene layers on the SiO_2_/Si substrate and to estimate the number of graphene layers through the intensity ratio of the 2D/G peaks [18,39]. In Figure 3a,b, a visual image of the graphene growth is shown next to and on top of the copper-strip for a selected mapping area of 150 × 100 μm^2^. In Figure 3a, it is possible to identify the general uniformity of the graphene growth throughout the mapped areas, while in Figure 3b the presence of the copper particles are clearly observed. Figure 3c,d show the Raman mapping of the intensity ratio of 2D/G peaks, for the same areas next to and on top of the copper-strip shown in Figure 3a,b. The average 2D/G ratio was 0.70 ± 0.05 and 0.50 ± 0.07 for Figure 3c,d, respectively. It is possible to estimate the number of graphene layers from the value of the 2D/G intensity ratio, which in our case corresponds to few layers of graphene [18,32,35]. However, other reasons such as the doping levels in the graphene layer can have an effect on this value (2D/G intensity), leading to an incorrect estimate of the number of layers [44].

### 3.2. SEM Analysis

**Figure 4:**
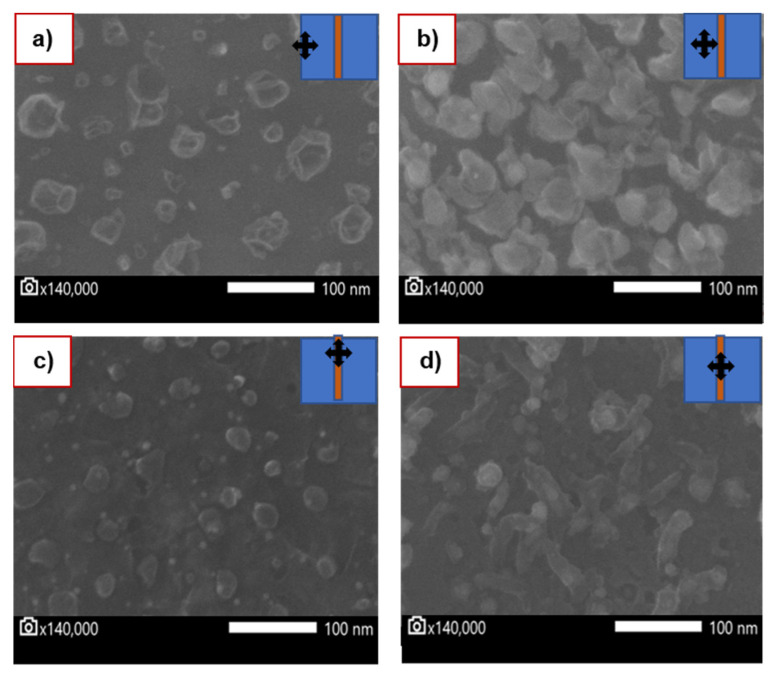
SEM measurements of the graphene growth on SiO_2_/Si substrate: (**a**,**b**) show the SEM image taken in two areas next to the copper-strip at 140,000×. Similarly, (**c**,**d**) show two areas on top of the copper strip at the same magnification. In all cases, crossed arrows represent the position relative to the copper strip where the image was taken.

Figure 4a,b show the SEM images taken in two areas next to the copper strip with a magnification of 140,000×. Figure 4a shows an area 8 mm from the copper film, while Figure 4b is an area closer (4 mm) to the copper strip. Similarly, Figure 4c,d show two different areas on top of the copper strip, upper and middle.

From the SEM images, it was possible to estimate the size of the graphene crystals from the scale bar to ca.100 nm. By measuring many crystals, we obtained an average size of 120 nm and a range of 100 to 140 nm for particles next to the copper strip, and smaller particles (35–120 nm; average size = 74 nm) on top of the copper-strip. At lower magnification (5000×), no copper particles were observed next to the copper film.

### 3.3. AFM Analysis

**Figure 5:**
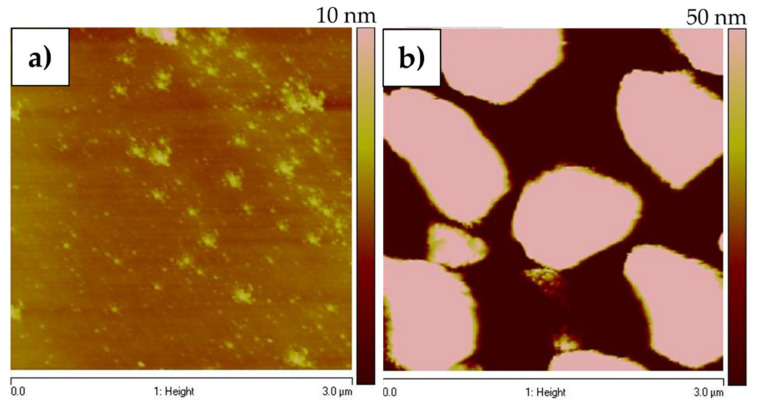
AFM measurements of the graphene growth on SiO_2_/Si substrate: (**a**,**b**) show the AFM images taken next to and on top of the copper-strip, respectively.

Figure 5 shows the AFM measurements for graphene growth on SiO_2_/Si substrate for both next to (cf. Figure 5a) and on top of (cf. Figure 5b) the copper-strip area, respectively. The copper grains were identified with an average height of 50 nm (Figure 5b) and uniform graphene layers were observed next to the copper strip with an average height of 5 nm (Figure 5a) corresponding to 6–12 graphene layers [18,46,47,48], supporting our calculations obtained from the Raman spectra. A nanocrystalline pattern was expected to be found, [36] however this was not identified because the deposited carbon material was composed of more than one layer of graphene. Nevertheless, two different morphologies were observed between areas on top of and next to the copper-strip.

### 3.4. EDS Analysis

**Figure 6:**
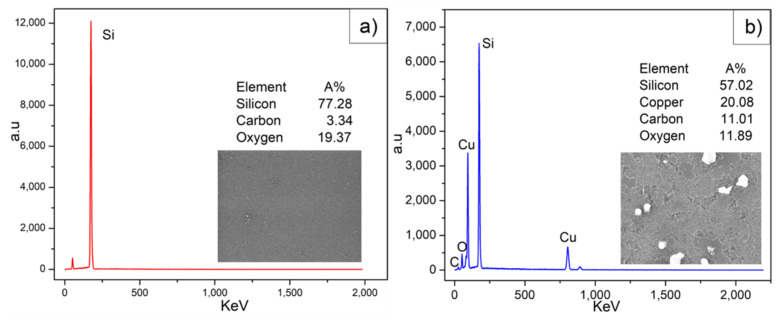
EDS spectrum of graphene on SiO_2_/Si substrate (**a**,**b**) shows the EDS spectrum next to and on top of the copper-strip, respectively.

A compositional analysis of graphene on SiO_2_/Si samples was done by EDS. In areas next to the copper strip (cf. Figure 6a), the following elements were identified (with their respective atomic concentrations): silicon (77.28%), oxygen (19.37%) and carbon (3.34%). In the EDS spectra on top of the copper-strip, the following elements were observed, silicon (57.02%), oxygen (11.89%), copper (20.08%) and carbon (11.01%) (cf. Figure 6b).

These atomic concentrations are consistent with the 2D/G intensity ratio in the Raman mapping experiment, where the lower values were found on top of the copper strip areas, indicating that more carbon atoms were deposited [35]. Although a higher carbon concentration was presented on top of the copper strip, a considerable percentage next to the metal film was identified. Additionally, no trace of copper was found next to the copper strip area, showing that there is graphene growth in metal-free areas.

### 3.5. XPS Analysis

**Figure 7:**
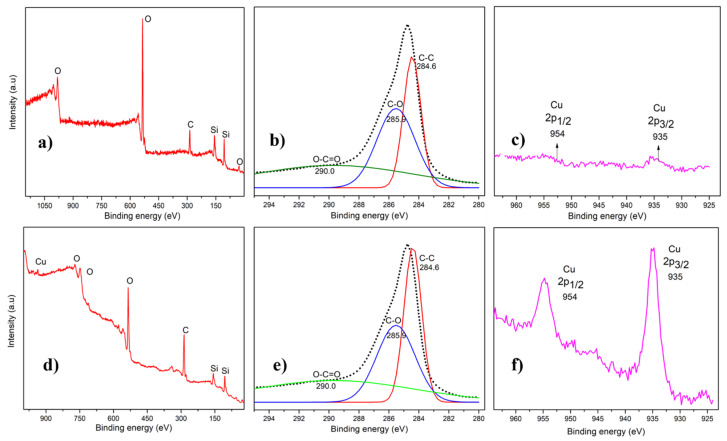
XPS measurements of the graphene growth on SiO_2_/Si substrate: (**a**) shows the XPS full composition spectra, (**b**) carbon peak after deconvolution and (**c**) the copper peaks taken next to the copper-strip. Then (**d**–**f**) represent the same but on top of the copper-strip.

XPS measurements were taken both next to and on top of the copper-strip. Figure 7a,d show the spectra of the elements found in both areas, respectively. The carbon 1s (C1s) peak was observed in both areas (Figure 7b,e). The raw data is shown on the dotted line and the solid lines represent the contribution of all the peaks after deconvolution. Contribution peaks were observed at 284.6 eV, 285.9 eV and 290.0 eV, corresponding to C-C, C-O and O-C=O respectively [38,49,50]. The presence of oxygen is confirmed in both areas in the XPS spectra (Figure 7a,d). The incorporation of oxygen most likely occurred after the graphene growth following exposure to air. The copper peaks (Cu 2p_3/2_: 930–937 eV and Cu 2p_1/2_: −954 eV) were observed on top of the copper-strip (Figure 7f), as expected. However, this metal shows a very small signal next to the copper-strip area (Figure 7c). Signals from other metals such as Fe (Fe 2p_3/2_: 706.7–710.9 eV), Co (Co 2p_3/2_: 778.1–780.2 eV) and Ni (Ni 2p_3/2_: 852.5–854.4 eV) were not observed on areas next to and on top of the copper-strip. The absence of other metals demonstrates that the graphene growth was either catalyst free or catalyzed by copper [25]. (Figure 7a,d).

The structural (Raman), morphological (SEM and AFM) and compositional (EDS and XPS) characteristics of the graphene on SiO_2_/Si substrate samples were measured. This characterization confirmed that this graphitic material grew over all areas of the SiO_2_ substrate at the nanocrystalline scale. The calculated grain size from Raman measurements was between 24.03 to 64.07 nm (next to the copper-strip); however, defects in the crystal due to phonon scattering may lead to an error in this estimate. These defects inside of the graphene nanocrystal were corroborated by the calculation of the inter-distance defect (Equation (2)).

**Figure 8:**
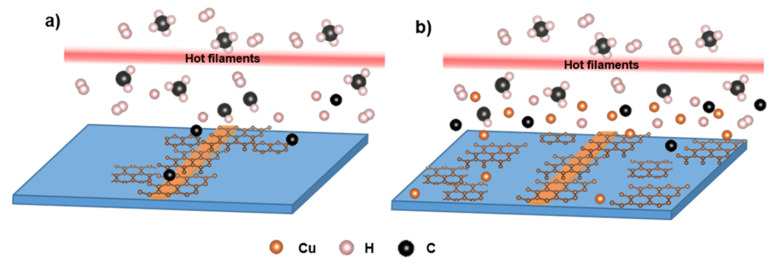
Schematic of the graphene growth mechanism on SiO_2_/Si substrate. (**a**) graphene migration from the copper-strip film and (**b**) the catalytic effect of the copper vapor to form graphene. In both figures, CH_4_/H_2_ molecules pass through the hot filaments prior to deposition. For more detail on the mechanism, see text.

The real size was confirmed through the images taken by the SEM technique where the particle size was in a range of 35 to 140 nm with an average of 120 nm (next to the copper-strip). The growth mechanism most likely begins with dehydrogenation of methane by the hot filament [18]. In the absence of copper, no graphene is observed, and therefore the growth must be catalyzed by the metal. This raises the question of whether the graphene is catalyzed on the metal film and migrates across the surface to cover the substrate (Figure 8a), or if the catalysis occurs due to vapor phase copper species above the surface (Figure 8b) [18,32,51,52,53]. If the vapor phase metal-catalyzed mechanism is operative, the expectation is that copper should be present across the substrate. While we do not see abundant amounts of copper next to the copper film, we cannot conclusively rule out the mechanism shown in Figure 8b because of the small signal observed in our XPS data. According to the growth distribution of graphene on the substrate we suggest that some crystals grew as migration from the copper-strip (Figure 8a), but some of the crystals next to the metal film were formed by the copper vapor catalyst effect (Figure 8b) [51,52] that is evaporated during the growth stage [31,51,52,53], leaving a small residual amount consistent with our XPS data.

## 4. Discussion

This study demonstrates, for the first time, a method to deposit polycrystalline graphene directly onto SiO_2_/Si by HFCVD, avoiding a complex graphene transfer process. In this method, a thin copper-strip of 0.3 cm × 2.0 cm was deposited in the middle of a 4 cm^2^ substrate, leaving most of the substrate surface free of this metal. The structural analysis was done by Raman spectra to verify the graphene growth characteristics. SEM and AFM images allowed us to determine the graphene’s topography on the SiO_2_/Si substrate. Additionally, copper residues were observed on top of the copper-strip areas, but these were not present in areas next to the metal. A compositional study was made through EDS and XPS measurements, indicating the presence of carbon in all samples and the virtual absence of copper in areas next to the metal-strip. This work demonstrates that the thin copper-strip deposited on the middle of the SiO_2_/Si enables the graphene growth over all the substrate. By eliminating the need for a mechanical transfer step in the device fabrication process, this accomplishment opens up the possibility of integrating graphene with currently available silicon device technologies. Further research, needed to continuously improve the quality of the graphene deposition, is ongoing in our laboratories. One approach is the reduction of the nucleation density [15,54,55,56] by modifying the methane and hydrogen gas flow rates that will allow an increment in the graphene crystal size and reduction of the point defects [55,56].

## 5. Conclusions

This work presents an approach to directly grow graphene on SiO_2_/Si by HFCVD, using the metal catalyzed method in a limited manner. The crystal size, structure, and inter-defect distance of the nanocrystalline graphene were estimated by SEM, AFM, and Raman measurements, respectively. EDS and XPS analyses confirmed the presence of graphene on SiO_2_/Si with negligible amount of copper in the area next to the copper strip. Our study allows the possibility of growing graphene directly on dielectrics without a transfer process and the opportunity to produce it on an industrial scale.

## Figures and Tables

**Table 1:** Methodologies to grow graphene on non-metallic substrates by CVD. Some growth parameters such as gas flow, temperature and carbon source are presented.

Method	CVD Type	Substrate	Pre-Growth Step	Carbon Source/ Temperature	References
**Catalyst-free**	Tube Furnace	SiO_2_ (0, 90, 300, 500 nm)/Si	H_2_ (70–160 sccm)/1060–1100 °C	CH_4_ (30 sccm)/1060–1100 °C	[27]
		SiO_2_ (300 nm)/Si	H_2_ (50 sccm) and Ar (1000 sccm)/1000 °C	CH_4_ (300 sccm)/1000 °C	[28]
	ECR plasma	SiO_2_/Si, quartz, and glass	Ar (5sccm)/400 °C	C_2_H_4_ (0.12 sccm) and Ar (0.12 sccm)/400 °C	[29]
**Metal-catalyzed**	Tube Furnace	Ni layer/silicon	H_2_ or He (400sccm)/ 900 °C	CH_4_ or C_2_H_2_ (50 sccm) and H_2_ (50 sccm)/900 °C	[30]
		Cu layer (60 nm)/SiO_2_ (300 nm)/Si	H_2_ (35 sccm)/1000 °C	CH_4_ (30 sccm) and H_2_ (20 sccm)/960 °C	[31]
		Cu layer (450 to 100 nm)/quartz, sapphire, SiO_2_ (300 nm)/Si, and fused silica	H_2_ (35 sccm)/1000 °C	CH_4_ (35 sccm) and H_2_ (2 sccm)/1000 °C	[32]
	Rapid heating plasma	Ni film (55 nm)/SiO_2_ (300 nm)/Si	CH_4_:H_2_ (9:1)/600–975 °C	CH_4_:H_2_ (9:1)/950 °C	[33]

## Data Availability

All data can be obtained from the corresponding author.
